# Materials, Electrical Performance, Mechanisms, Applications, and Manufacturing Approaches for Flexible Strain Sensors

**DOI:** 10.3390/nano11051220

**Published:** 2021-05-05

**Authors:** Fei Han, Min Li, Huaiyu Ye, Guoqi Zhang

**Affiliations:** 1 Institute of Future Lighting, Academy for Engineering and Technology, Fudan University, Shanghai 200433, China; hanfei6090@163.com (F.H.); lm_ss@fudan.edu.cn (M.L.); 2 Shenzhen Institute of Wide-Bandgap Semiconductors, Shenzhen 518055, China

**Keywords:** flexible electronics, strain sensors, flexible materials, implantable sensors

## Abstract

With the recent great progress made in flexible and wearable electronic materials, the upcoming next generation of skin-mountable and implantable smart devices holds extensive potential applications for the lifestyle modifying, including personalized health monitoring, human-machine interfaces, soft robots, and implantable biomedical devices. As a core member within the wearable electronics family, flexible strain sensors play an essential role in the structure design and functional optimization. To further enhance the stretchability, flexibility, sensitivity, and electricity performances of the flexible strain sensors, enormous efforts have been done covering the materials design, manufacturing approaches and various applications. Thus, this review summarizes the latest advances in flexible strain sensors over recent years from the material, application, and manufacturing strategies. Firstly, the critical parameters measuring the performances of flexible strain sensors and materials development contains different flexible substrates, new nano- and hybrid- materials are introduced. Then, the developed working mechanisms, theoretical analysis, and computational simulation are presented. Next, based on different material design, diverse applications including human motion detection and health monitoring, soft robotics and human-machine interface, implantable devices, and biomedical applications are highlighted. Finally, synthesis consideration of the massive production industry of flexible strain sensors in the future; different fabrication approaches that are fully expected are classified and discussed.

## 1. Introduction

Flexible and wearable electronics have recently attracted wide attention due to their great potential applications in real-time human health monitoring systems (e.g., detection of human motions [1,2,3,4], heart-beat [5,6], blood pressure [7,8,9], and beyond [10,11,12]), human-machine interfaces [13,14,15] (e.g., flexible sensors work as a medium and dialogue interface for the transmission and exchange of information between humans and machines), and implantable devices [6,16,17,18] (e.g., transmit the sense of skin touch information to the brain by using electronic skin, and prosthesis was controlled by the cerebral cortex with 3D microelectrodes, etc.). The synchronized delivery and control of the signal from human body to detector or actuator are convenient, expeditious, effective, and accurate compared with traditional rigid conducting and semiconducting materials based on smart devices [19,20]. Besides, the excellent stretchability [21,22], transparency [23,24,25], wearability [7,26,27], and biocompatibility [28,29,30] endows flexible and wearable electronics with alluring prospect, which the functions infinitely tend to real human skins or beyond [31,32,33].

Flexible strain sensors, one of the vital members in the flexible and wearable electronics family, have been rapidly developed in recent years by emerging materials and technologies. The physiological activity signals can be covert into different visual electrical signals in the form of signal transduction by flexible strain sensors [34]. With extensive research on flexible strain sensors from all over the world, great progresses have been made in materials preparation and application scenarios. Thus, a wide variety of review works recently have been done to summarize the latest advances in flexible strain sensors. For example, the emerging materials-based including carbon nanotube, [35] graphene [36], and metal nano-wires/particles [37], etc., reviews on flexible strain sensors are concluded, respectively. Moreover, some systematic summaries were made to conclude the progress of flexible strain sensors from a comprehensive perspective [38,39]. However, with the fast growth of flexible strain sensors filed, there is a looming trend and urgent requirement for their large-scale manufacture for practical application with a degree of commercial value and practical value. From this point of view, we note that the integrated review work equipped with the latest materials preparation, computational simulation, and especially manufacturing approaches have not been discussed systematically.

**Figure 1:**
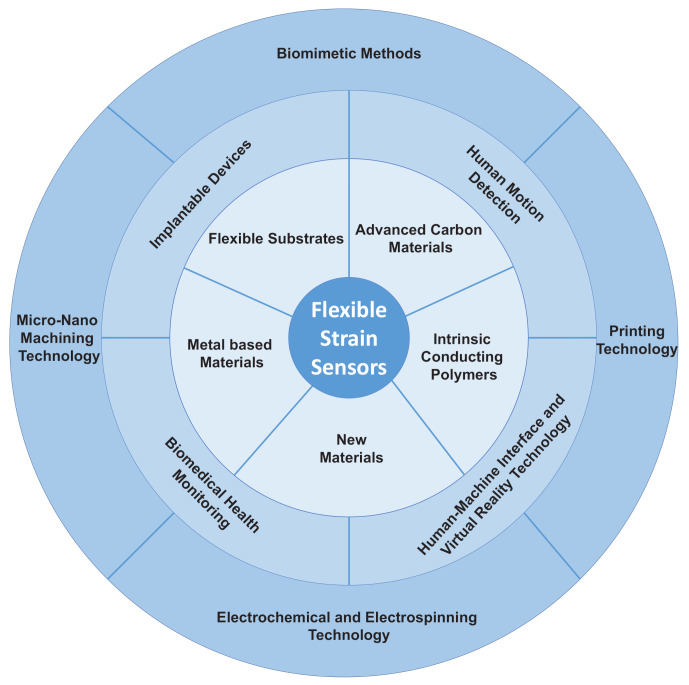
Schematic diagram of the mainly materials, applications, and manufacture approaches for flexible strain sensors.

Therefore, in this review, the latest advances in flexible strain sensor over recent years from the material, application, and manufacturing strategies are summarized (Figure 1). Firstly, the critical parameters measuring the performances of flexible strain sensors and materials development contains different flexible substrates, new nano- and hybrid- materials are introduced. Then, the developed working mechanisms, theoretical analysis, and computational simulation are presented. Next, based on different material design, diverse applications including human motion detection and health monitoring, soft robotics and human-machine interface, implantable devices, and biomedical applications are highlighted. Finally, synthesis consideration of the massive production industry of flexible strain sensors in the future; different fabrication approaches that are fully expected are classified and discussed.

## 2. Critical Parameters of Strain sensors

### 2.1. Stretchability and Hysteresis

Stretchability is a basic parameter to evaluate the performance of most of resistive-type flexible strain sensors [33,38] (the change of electrical resistance as a function of the applied strain, typically composed of electrically conductive sensing fillers coupled with flexible substrates). Due to the poor stretchability of inorganic conductive nanomaterials, flexible, and stretchable substrates (e.g., polydimethylsiloxane (PDMS), [40] polyurethanes (PU), [41] rubbers and Ecoflex, [42,43] leather, [44,45] etc.) are frequently selected to enhance the stretchability and flexibility of strain sensors. Design of materials structure is an effective way to improve the stretchability of conductive nanomaterials and stretchable polymer composite materials-based flexible strain sensors. Net-shaped structures, [46] spring-like structures, [47,48] wavy structures [48], and island-bridge structures [49] have been developed for the fabrication of highly stretchable strain sensors because their excellent structure accommodation when external strain is applied. Besides, tractable materials (e.g., carbon nanotubes (CNTs) [50,51] and metal nanowires (NWs) [21,52]) with appropriate processing method (e.g., electrospinning [53] and cross-stacking aligned ribbons [54]) also results in the stretchable structures. In addition, developing new intrinsic conductive polymer [55,56,57] with better stretchability is another direct way for flexible and stretchable strain sensors.

When flexible strain sensors are stretched under larger strain, hysteresis becomes the main effect factor of the performance for resistive-type flexible strain sensors. The electrical characteristics of the sensors cannot restore timely during the dynamic loading-unloading process [38,58]. According to the reported works, resistive-type sensors are more susceptible to hysteresis effect than capacitive-type sensors (the change of capacitance as a function of the applied strain, typically composed of a highly compliant dielectric layer sandwiched between a pair of stretchable electrodes), due to different work mechanisms [58]. The variation of the overlapped area between two electrodes for capacitive-type sensors are smaller than the geometric deformations for resistive-type sensors under tensile strain. Moreover, the viscoelasticity of polymers (such as PDMS) and the interaction between conductive nanomaterial fillers and polymers are two major factors that caused hysteresis. The viscoelastic property of polymers leads the inherent hysteresis for the sensors, and the hysteresis effect can be ignored when sensors under small strains. But with the applied strain increased, the weaker deformation recovery capability of polymers and the slides of the inside conductive nanomaterials (such as CNTs, [59] graphene [60], and metal NWs [61]) results in higher hysteresis behavior. Thus, some of works are focus on the interaction between conductive nanomaterial fillers and polymer materials for high performance flexible strain sensors [62,63,64].

### 2.2. Sensitivity and Linearity

The sensitivity, also known as gauge factor (GF), of flexible strain sensor is defined as the slope of the relative change of electrical signal versus applied strain. According to the definition of GF, it is generally calculated by GF = (ΔR/R_0_)/Δε for resistive-type sensors and GF = (ΔC/C_0_)/Δε for capacitive-type sensors, where ΔR and ΔC is the variation of relative resistance and capacitance change, respectively, R_0_ and C_0_ represent the electrical resistance and capacitance in relaxed states, respectively, and the ε is the applied strain of sensor. Different GF values reflect the different sensitivity of the sensors under applied strains. Among the reported works, both two or more GF values exist in one sensor within the limited strains, [65,66,67] but also one GF value with the increased strain in a sensor [68,69]. And these are tightly associated with the materials and mechanisms of the sensors system.

The response electrical signal versus applied strain with a settled slope within applied strains is the significant sign called linearity performance for flexible strain sensors. It is a vital parameter to analyze the stability and predict corresponding status of the flexible sensor. However, most of resistive-type strain sensors show nonlinearity due to the unsynchronized inside structures changes, such as graphene, [70] CNTs, [71] and silver NWs- (AgNWs) [72] based composites. On the contrary, excellent linearity is commonly expressed on capacitive-type sensors but with low sensitivity [73]. Furthermore, to realize highly sensitive strain sensors with high stretchability and linearity is a tricky matter. Therefore, further researches to balance those three parameters are indispensable.

### 2.3. Response Time and Durability

Response time reflects the response speed of the strain sensors to achieve 90% steady states. There are two factors affecting response time, the viscoelastic property of polymers and the contact stability between conductive materials and electrodes. Generally, the response time varies from tens of milliseconds (mostly for capacitive-type strain sensors) to several hundred milliseconds (mostly for resistive-type strain sensors). Accordingly, recovery time also showed extremely short property on capacitive-type strain sensors, while relatively longer time on resistive-type strain sensors due to the untimely recovery of inside conductive structure.

**Figure 2:**
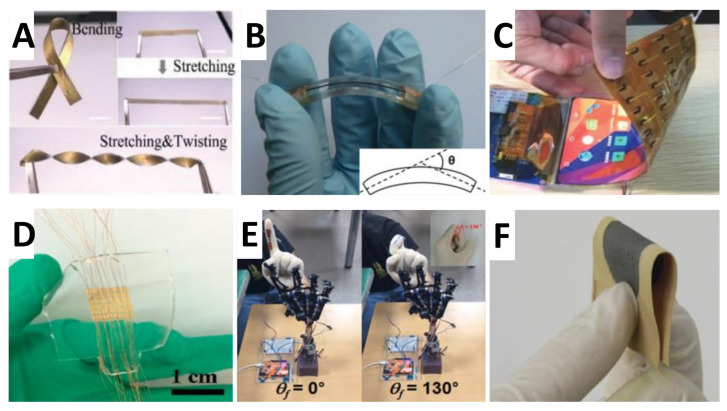
Various flexible, skin-mountable, and wearable strain sensors. (**A**) Photograph of an Au/PDMS film during different deformations. Scale bars: 1 cm. Reproduced with the permission from [76], copyright the Wiley Online Library, 2019. (**B**) Photograph of the bending sensor. Reproduced with the permission from [77], copyright the Wiley Online Library, 2015. (**C**) The photoimage of bendable interactive surface. Reproduced with the permission from [78], copyright the Wiley Online Library, 2020. (**D**) Optical image of the strain sensor array (5 × 5). Reproduced with the permission from [79], copyright the ACS Publications, 2016. (**E**) Remote control of a robotic finger. Reproduced with the permission from [80], copyright the ACS Publications, 2018. (**F**) Pictures of the conductive cotton fabric. Reproduced with the permission from [81], copyright the American Carbon Society, 2017.

Durability was introduced to measure the long-time service performance under dynamic stretching-releasing cycles of flexible strain sensors. The nature characteristics of sensors and the operational environment are two points to determine the sensors lifetime. There are no specific defined requirements for how many precise cycles of the strain sensors should reached, but commonly more than 5000 cycles [74] (some even more than 10,000 cycles [75]) are performed among the reported flexible strain sensors. And the demonstrated instability output signals during the stability test are caused by the inside slides between conductive nanomaterials and polymers, and the irreversible defect occurred in the cycles process. Figure 2 illustrates several flexible, skin-mountable, and wearable strain sensors made of functional nanomaterials and stretchable polymer composites.

## 3. Materials Development

**Figure 3:**
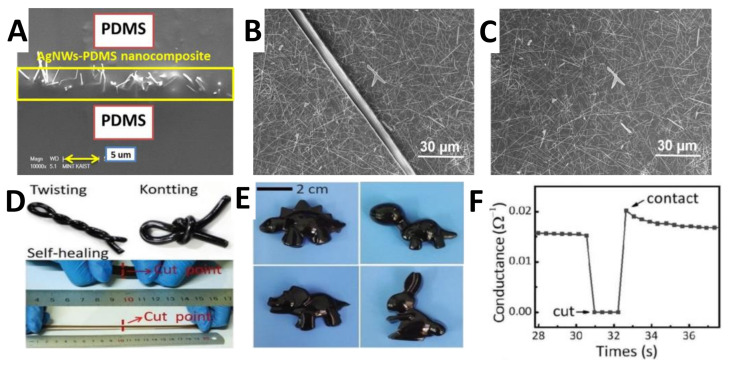
(**A**) Cross-sectional SEM image of the sandwich-structured strain sensor. Reproduced with the permission from [91], copyright the ACS Publications, 2014. (**B**,**C**) SEM images of (**B**) the scratched and (**C**) the sunlight healed surface of the AgNW/PU composite film. Reproduced with the permission from [92], copyright the Royal Society of Chemistry, 2019. (**D**,**E**) Design and fabrication of multifunctional ionogel nanocomposites. Reproduced with the permission from [93], copyright the Wiley Online Library, 2019. (**F**) Time dependence of conductance and healing speed of the ionogel nanocomposites. Reproduced with the permission from [93], copyright the Wiley Online Library, 2019.

The basic materials composition for flexible strain sensors cover flexible and stretchable support substrate and conductive nanomaterials. To prepare strain sensors with high flexibility and stretchability (even with self-healing function [82]), polymers including PDMS, [83] PU, [84] polyethylene terephthalate (PET), [85] rubbers, [86] Ecoflex, [87] polyamide, [88] polytetrafluoroethylene (PTFE), [89] polyvinyl chloride (PVC) [90], etc., were employed as different support substrates or flexible framework structure. Furthermore, nanomaterials with excellent conductivity and mechanical properties (including advanced carbon materials, nano-metal materials, and the hybrids) have been widely utilized for the sensor fabrication (Figure 3). In addition, conductive polymers as a special material also have drawn much attention. Thus, we will introduce the development of fore-mentioned materials-based sensors specifically in this part.

### 3.1. Flexible Substrates

Different with traditional strain sensors, which are silicon- and silicon oxide-based rigid and fragile materials, support substrates with excellent flexibility and stretchability are indispensable qualities for flexible strain sensors. For this purpose, flexible polymers are selected as the adaptive candidate and studied extensively for support substrates.

PDMS, high molecular organosilicon compound, has been extensively employed as flexible support substrate for sensors fabrication on account of the advantages as follows: (1) low young’s modulus leading its excellent flexibility and stretchability, (2) corrosion-resistance performance with good transparency and stability in a wide range of temperatures create the reliability, (3) skin-mountable and non-allergic properties make the smooth road towards the wearable electronics, (4) facile fabrication method and controllable designed geometric structure bring the wide promotion. For example, a sandwich structure PDMS/AgNWs/PDMS- (AgNWs thin film embedded between two layers of PDMS) based strain sensor was reported with high stretchability up to 70% and controllable linearity sensing performance [91]. Also, Ag mesh/PDMS strain sensor with honeycomb lattices and multiple domains as sensitive unit was introduced for the sensitive and transparent sensor applications [94].

PU is another flexible support substrate with excellent environmental stability and mechanical property. PU composites-based strain sensors were reported frequently by different preparation method for high sensitive, low detect limitation and long-term cyclic stability sensing operations. Moreover, PU sponges with flexible skeleton structure were demonstrated for strain sensors [89,95,96]. In addition, other optional flexible film and polymers, such as PET, rubbers, and Ecoflex, etc., were employed for the flexible strain sensors manufacture.

Besides, it is worth mentioning that polymers with self-healing function are widely applied to multi-functional strain sensors fabrication recently, which is a vital feature mimic human skin and greatly improved the sensors service life and stability. A sunlight self-healable transparent strain sensor was demonstrated by Zhang et al. PU using based composites combined with rational structure design [92]. The fabricated sensors possess cycling durability and can be self-healed with the stimulus of sunshine. Besides, an ionogel nanocomposite as strain sensor was presented by Suo et al. with excellent self-healing (>95% healing efficiency) and ultra-high stretchability (2000%) and exhibited a great potential to detect large deformation objects with high sensitivity [93].

### 3.2. Advanced Carbon Materials

Advanced carbon materials represented by graphene, graphite, CNTs, carbon black (CB), and other carbon materials derived by natural biomaterials have been used as sensing units for flexible strain sensors due to their excellent conductive ability and mechanical property. Thus, in the section, we will discuss the recent progress on flexible strain sensors made by advanced carbon materials in detail.

Since the 2010 Nobel prize in Physics awarded to the groundbreaking work, “two-dimensional (2D) material graphene”, graphene has been the beloved candidate for various applications, which naturally includes flexible electronics. Indeed, the outstanding electronic properties of ultra-high intrinsic mobility and mechanical properties with fracture strains of ca. 25% and Young’s modulus of ca. 1TPa make it highly suitable for flexible electronics. High-quality and high-homogeneous graphene-based strain sensors by chemical vapor deposition (CVD) were reported exhibiting good transparent and linearity response, infinitesimal detect limitation (e.g., as low as 0.1% strain [97]), and rarely extremely high sensitivity (e.g., with GF of 1037 at 2% strain [98]). Besides, cyclically stable, highly stretchable, and sensitive graphene-based strain sensors were fabricated through solution oxidation–reduction process [95]. It can be seen that to balance the sensitivity, stretchability, and linearity parameters integrated into a graphene-based strain sensor remains the problem to overcome.

CNTs with diverse formats (such as CNT fibers, arrays, and films) have been extensively studied as the promising materials for high performance flexible strain sensors, due to their large aspect ratio, excellent electrical conductivity, and flexibility. A multi stimuli sensing multi-walled CNTs-PDMS composites-based resistive-type sensor by Ko et al. was reported with high GF of 9617 and 120% strain ranges but non-linearity performance [99]. Conversely, a highly stretched (up to 300% strain) with good linearity but lower sensitive (GF of 1) capacitive-type sensor was demonstrated by using single-walled CNTs-PDMS composites [100]. Through the comprehensive view of the CNTs based sensors performances, to realizing high reproduction quality and controllable structures with low-cost strategies are still barriers for their further development.

By comparison, other cheaper and available carbon materials, such as carbon black, graphite, and natural-biomaterial-derived carbon materials are also suitable raw materials for flexible strain sensors fabrication. For instance, CB-based composites for strain sensors have been studied by blending modification with polymers and conductive printing inks with additional components [73]. For graphite, facile pencil-writing method was developed for sensors preparation with better sensitivity under tiny strain; [101] and printing technology also introduced for graphite-based strain sensors [102]. Besides, carbonized materials from thermal treatment resulted natural biomaterials [103] (e.g., silk, [104] corncobs, [105] cotton, [106] crepe paper, [107] mushrooms, [108], etc.) for strain sensors have been demonstrated due to their low-cost, renewable, and eco-friendly capabilities. For example, nano-sponge, silk fabric, and cotton derived carbonized materials with designed structures were reported for high performance flexible strain sensors with high stability (>1000 cycles), wide sensing range (from 0% to more than 500% strain), and better sensitivity (GF of 25 in strain of 0%–80% and that of 64 in strain of 80%–140%); and many other natural-biomaterial-derived carbon materials with unique structures for flexible strain sensors can be studied further in the long run.

### 3.3. Metal Based Materials

Besides the advanced carbon materials, metal materials, such as metal nano-wires/particles/flakes, and liquid metal, which are hot options, have also been used in flexible strain sensors. In the part, the advancements in silver, gold and copper-based nanomaterials and hybrid materials for strain sensors applications will be elaborated. Moreover, the recent emerged Ga-based liquid metal for flexible strain sensors are also highlighted.

**Figure 4:**
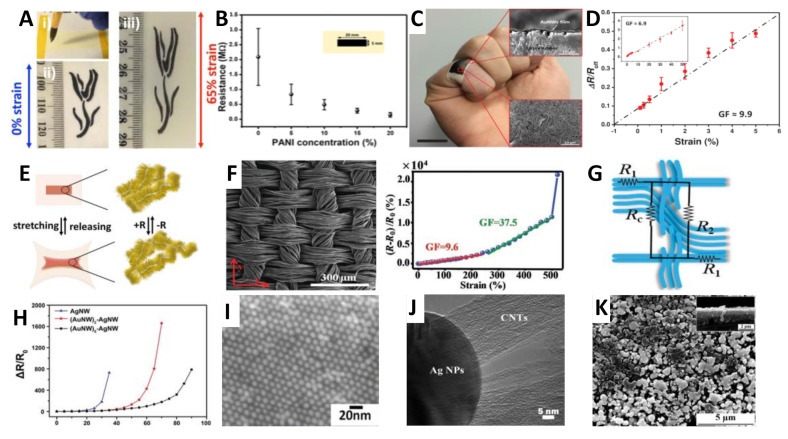
(**A**) Photograph of the direct-writing process for sensors fabrication. Reproduced with the permission from [110], copyright the ACS Publications, 2015. (**B**) Plot of sheet resistance of AuNWs/PANI film. Reproduced with the permission from [110], copyright the ACS Publications, 2015. (**C**,**D**) Photographs of a strain sensor ring attached on the little finger while bending (scale bar: 1 cm), and electrical resistance changes under various strains. Reproduced with the permission from [111], copyright the Wiley Online Library, 2015. (**E**) The schematic of the AuNWs-functionalised fibers sensor. Reproduced with the permission from [112], copyright the Wiley Online Library, 2019. (**F**,**G**) SEM of the carbonized silk fabric, relative change in resistance of the strain sensor versus the applied strain. Schematic illustration showing the resistance model of an elementary unit. Reproduced with the permission from [104], copyright the Wiley Online Library, 2016. (**H**) Relative resistance changes as a function of the applied strain for different samples. Reproduced with the permission from [113], copyright the Wiley Online Library, 2018. (**I**) Successive SEM zoom-ins of the assembly of 14 nm gold nanoparticles between the electrodes. Reproduced with the permission from [114], copyright the Royal Society of Chemistry, 2018. (**J**) The AgNPs@CNTs contact interface. Reproduced with the permission from [115], copyright the ACS Publications, 2016. (**K**) The surface of the nanocomposite embedded onto PDMS. Reproduced with the permission from [116], copyright the Elsevier B.V., 2015.

Metal nanowires offer several attributes for flexible strain sensors (Figure 4), including distinguished electric conductivity, thermal and mechanical flexibility, good transparency, and especially high aspect ratio (AR) characteristics. AgNWs, with the highest electrical conductivity among all the metal materials, have been researched extensively for flexible electronics assisted by diverse synthesis methods (e.g., hydrothermal method, electrochemical technique, and the polyol approach). To combine with flexible substrates for strain sensors, spray coating, ultrafiltration, and printing techniques were developed to explore the cost-effective and mass production method. The stretchability of AgNWs-based strain sensor could be improved by pre-strain method resulted wavy structure, [109] and the higher conductivity of that obtained by introduce long NWs and solder the junctions to lengthen percolation paths and reduce NW junctions, as well as reduce the contact resistance respectively. In addition, a sandwich PDMS/AgNWs/PDMS structure strain sensor was reported with a high linearity and especially negligible hysteresis within 40% strain by Park et al. group [91].

Gold NWs (AuNWs) are particularly attractive for flexible electronics, due to their outstanding biocompatibility, environmental stability, as well as excellent electrical conductivity. According to the reported works, ultrathin AuNWs show serpentine structure when their dimensions under certain value (<5 nm in width, with AR of >10000), which is a vital property when applied to flexible electronics. For example, Cheng’s group has done a mountain of works on AuNWs fabrication and application [111,112]. The polyaniline microparticles doped AuNWs films were fabricated with 10 times enhancement in conductivity and ∼8 times improvement in sensitivity in comparison to the sensors without PANI particles [110]. In addition, AuNWs–AgNWs hybrid materials were also explored for high performance strain sensors. Li et al. reported a binary metal nanowire percolation network through the collaboration of continuous AuNW backbone network and thin AgNW network to realize highly transparent strain sensors with extraordinary sensing ability in a wide range of strains [113]. The resulted strain sensors exhibited a wide sensing range up to 90%, high GF values from 12 at 5% to 2370 at 70%, high transmittance of 86%, and prominent cyclical stability (1000 times). Similarly, recently reported Ag–Au nanocomposites composed of ultra-long gold-coated silver NWs in an elastomeric block-copolymer matrix possess a fully tensile strain of 840% simultaneously an optimized conductivity of 41,850 S cm^−1^ [117]. The two examples fully interpreted the excellent synergistic effect of AuNWs–AgNWs hybrid materials; and the distinguished biocompatibility and chemical inertness make AuNWs as ideal candidate for implantable and flexible electronics.

Despite the outstanding performance achieved by AgNWs- and AuNWs-based materials, the limited storage and high cost constrained their large area applications when considerating commercial promotion in the future. Thus, copper NWs (CuNWs) came into our vision because its high intrinsic conductivity (only second to silver), high abundance (1000 times more abundant than silver), and low cost (100 times cheaper than Ag and 6000 times cheaper than Au) properties, which were summarized in our previous review work. Based on those advantages, CuNWs/PDMS composites were developed by different structure design (e.g., wrinkled structure and helical structure) and process treatment (e.g., spinning coating, template-assisted method, and printing technology) for flexible strain sensors [118]. Another device includes microchannels filled with a PEDOT:PSS solution and a conductive nanowire network (AgNWs, CuNWs, and CNTs) embedded at the bottom of the microchannel was explored with highly sensitivity of GF values of 2000, 1687, and 800, respectively [119]. However, the un-economical and un-efficient synthetic methods, harder controlled structures, and especially frangibility to oxidation of CuNWs are the main stumbling blocks for its further applications; and more efforts are urgently demanded to seek for efficient approaches and to go deeply into its growth mechanism. For anti-oxidation purpose, design of core–shell hetero-structures and introduce reductive agent into the materials are major concern solutions at the current stage [37,118]. It is worth mentioning that besides the fore-mentioned metal NWs, metal oxide NWs (e.g., ZnO NWs [120,121]) were also explored for multifunctional sensors fabrication.

Apart from the one dimensional metal NWs, zero dimensional (0D) metal nanoparticles (NPs) (NPs’ size vary from 1 to 100 nm), such as AgNPs, AuNPs, and the hybrids have been successfully adopted on flexible strain sensors. For example, AgNPs combined with CNTs, [116] AgNWs, [122] graphene, [123] epoxy natural rubber [124], and PDMS [125] were developed for strain sensors with satisfactory performances. Grisolia et al. has reported colloidal AuNPs based capacitive strain sensors with tunable sensitivity; [114] and the resultant sensors could detect ultralow change of 0.5% strain. To prove the synthesis of plasmonic-based microcapsules, an approach based on controlled dense packing of AuNPs at the surface of emulsion droplets by tuning the charge of the nanoparticles, was demonstrated for strain sensors [126]. As an additional attribute, NPs with different shapes (e.g., sphere, rectangle, hexagon, star, and branch-like outlines) were synthesized and have great potential applied to flexible electronics.

**Figure 5:**
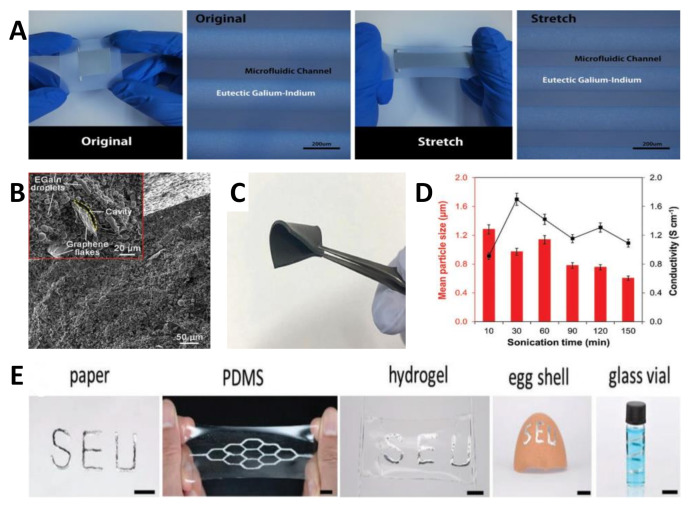
(**A**) Photograph of the liquid metal based super-stretchable sensor with no applied deformation being stretched in a direction parallel to the channel. Reproduced with the permission from [128], copyright the Springer Nature, 2019. (**B**–**D**) SEM images of the cross section of the composite material; photos show the flexibility of the composites; mean value of droplet size distribution and slurry conductivity as a function of sonication time. Reproduced with the permission from [127], copyright the Wiley Online Library, 2020. (**E**) Photographs of the patterned LM on different substrate including planar substrates (e.g., paper, PDMS, hydrogel) and curved surfaces of the eggshell and the inner wall of the glass vial. Scale bars: 10 mm. Reproduced with the permission from [129], copyright the Wiley Online Library, 2019.

Gallium-based liquid metal (LMs), emerged soft materials and with melting points lower than room temperature, are actively used in the field of flexible electronics because of their high electrical conductivity (3.4 × 10^6^ S m^−1^ for gallium–indium alloys) and extreme stretchability (>600% strain) without conductivity loss. To get composites without reduced integrity (Figure 5), graphene flakes incorporated with eutectic gallium–indium (EGaln) LMs in a PDMS matrix was developed with high conductivity values ranging from 8.5 to 39.0 S cm^−1^; [127] and the introduced non-functionalized graphene flakes were beneficial to the macro and nano-cavities filled by LMs. For flexible strain sensors, LMs micro- and nano- NPs embedded into polymers is a direct and effective way to overcome the limited mechanical stretchability and fragile conductive paths characters based on solid metal NPs and the hybrid. For example, Ecoflex microfluidic assembly filled with EGaln LMs sensor was prepared with an ultimate strain up to 550%, and the applications on human motions detections were conducted [128]. However, the fabrication process was complicated. For easy approaches, a simple method of direct patterning of LMs on various substrates using magnetic field was reported for flexible sensors; [129] and this method is compatible with non-modified substrate and nonplanar substrates. Overall, although LMs based flexible strain sensors have made some progress, the further and deeper property study of the LMs itself and the controllable synthesis specific sized LMs droplets are demanding for prompt actions. In addition to above-mentioned metal materials, MXene materials (a new class of two-dimensional transition metal carbides and carbonitrides) and third generation semiconductor materials were also researched extensively for flexible strain sensors [130,131,132,133,134,135,136].

### 3.4. Intrinsic Conducting Polymers

Besides the doped and modified polymers with better electrical conductivity discussed in part 3.1, there exists a kind of intrinsic conducting polymer, which also have been widely explored in the field of flexible electronics. Poly(3,4-ethylenedioxythiophene) (PEDOT), polyaniline (PANI), and polypyrrole (PPy) are three main π-conjugate polymers with intrinsic conductivity. Therefore, we will discuss the recent progress of those conductive polymers and the hybrid-based flexible strain sensors and beyond in this part.

PEDOT is a polymer with excellent conductivity (up to 300 S cm^−1^) due to the presence of PF_6-_ counter-ion but poorer solubility. To increase its solubility, negatively charged and water-soluble poly(styrenesulfonate) (PSS) was introduced to foam PEDOT:PSS, which PSS work as a stabilizer and aqueous dispersion carrier for PEDOT. The excellent electrical conductivity and water solubility of PEDOT:PSS make it suitable for flexible strain sensors. For example, a highly sensitive (GF of 280 at 0.6% strain) with lower detect limitation (<0.2% strain) strain sensor was reported consisting of a 150 nm thick dimethylsulfoxide-doped PEDOT:PSS sensing layer and an elastic fluorosilicone rubber substrate [137]. The widening of its microcracks and the disconnection of the PEDOT:PSS film contribute to the ultrasensitive sensor. Another PEDOT:PSS composites were manufactured by electrospinning method for strain sensors [138]. To increase the stretchability, doping is a good route, for instance, 1-ethyl-3-methylimidazolium tetracyanoborate was added to the PEDOT:PSS solution, and resulted in a conducting polymer with a high conductivity (>1000 S cm^−1^) up to 50% strain [139]. For better performance, J. Lipomi et al. reported the use of a PSS-based block copolymer to achieve stretchable PEDOT:PSS, which exhibited a higher toughness (up to 10.1 MJ m^−3^) and stretchability (up to 128%) [140]. However, the electrical conductivity was reduced by this method. Differently, Bao el al. demonstrated a highly stretchable and conductive PEDOT film (the conductivity over 3100 S/cm and over 4100 S/cm under 0% and 100% strain, respectively) with high cycling stability (the conductivity maintained at 3600 S/cm even after 1000 cycles to 100% strain) by incorporating ionic additives-assisted stretchability and electrical conductivity enhancers [141]. In addition, PEDOT- and PEDOT:PSS-based hydrogels, as other types of conducting polymers, also caused extensive concern in the flexible electronics area [142,143].

PANI is another representative conducting polymer due to its unique high environmental stability and multiple applications. For strain sensors, PANI commonly developed with alternative substrates and materials forming hybrid sensing units. Graphene, [144] AuNWs, [110] PDMS, [145] phytic acid [146], and polyacrylic acid [147] were employed with PANI-based composites for flexible strain sensors, and have revealed large tensile deformation (>30%, some even >1000%), good sensitivity and linear response (R^2^ >0.9994), as well as widespread applications. Specially, a free-standing 3D PANI foam with micro-cracked structure was fabricated by template-assisted method and electro-polymerization technique [145]. The prepared piezoresistive sensor possess multifunctional sensing properties, including for strain and pressure stimulate. Likewise, conductive PPy hybrid strain sensors were proposed combined with different materials. Zeng et al. reported PPy doped conductive polymer composites by a facile solution-casting method for self-healable flexible sensors [148]. And the incorporation of PPy played a vital role for the sensor mechanical strength. More additional studies were working on the in situ polymerized PPy in the sensing system. For example, Wang’s group demonstrated in situ polymerized PPy/PU elastomer sensor for waistband-like human breath detector use but with sensitivity [57]. Besides, a weavability and multimodal mechanical sensing fabric like sensor was designed by winding graphene oxide-doped polyacrylonitrile nanofiber yarns with in situ polymerized PPy on elastic yarns [149]. The sensors unit had high sensitivity (GF≈68), wide pressure-sensing range, and excellent repeatability (over 10,000 cycles). In order to witness the performance of different materials-based flexible strain sensors intuitively, the summarized data are shown in Table 1.

## 4. Working Mechanisms and Computational Simulation Analysis

Resistive-type and capacitive-type are two kinds of developed flexible strain sensors based on different materials, structures, and work mechanisms. Just because of this, several working mechanisms including geometric structure variation, piezoresistive effect, disconnection and micro-crack propagation mechanism, and tunneling effect have been introduced for the illustration of sensors working theory. To match up with the mentioned principles, computational simulation analysis came up for the deeper discovery of dynamic procedures and further behind that.

### 4.1. Geometric Structure Variation

For resistive-type strain sensors, the length increases in tensile direction and the dimensional shrinkage in the cross-sectional area by the applied external strain causing the resistances signals changes according to the equation R = ρL/A, where ρ, L, and A represent the electrical resistivity, the length, and the cross-sectional area of the material, respectively. For example, the effect of geometry changes for natural-based active materials on the sensing behavior of strain sensors has been explored by Souri et al. [168] When it comes to capacitive-type strain sensors, the loaded strain resulting in decreased thickness of the dielectric layer and the change of the capacitive area, which finally leads to the capacitance changes of the sensor. While the capacitance changes exhibit stable linearity versus applied strain relative to the resistive-type sensors, the linear interval was limited by the exerted strain. Interestingly, J. Cohen’s group illustrated the general mechanism by the fabricated CNTs and silicone based capacitive-type sensor and demonstrated its linearity using as angle transducer for four-bar linkage [169].

To figure out the relationship between desired properties and the effects of nano-materials, Ahn et al. proposed a simulation method for conductive NPs and NTs composite using the Lennard-Jones potential model and the voter model [170]. According to this method, the distribution of nanocomposites in the boundary conditions was first optimized utilizing Lennard-Jones potential model. After that, the electrical conductivity of the stain sensors was assessed by counting average attachment among nanomaterials under strain, cooperating with validated value by fabricated strain sensor with specific composition ration. Finally, the author confirmed that the sensor performance primarily dominated by the diameter of NPs, and the diameter of NTs, can be ignored. Besides, the largest change of electrical conductivity with the applied strain appears on small and uniform nanomaterials.

### 4.2. Piezoresistive Effect

Piezoresistive effect is defined as resistance changes of the materials versus the external mechanical deformations caused by the applied strain. According to its definition, the relative change of resistance by piezoresistive effect mainly contains two parts of compositions, which are the variation of geometric effect and the intrinsic piezoresistivity of materials, and can be written as ΔR/R_0_ = (1 + 2υ)ε + Δρ/ρ, where ε is the applied strain, (1 + 2υ)ε and Δρ/ρ represent the two compositions, respectively. While piezoresistive effect for metal and metal-alloy is relatively smaller on the increased resistance, the piezoresistivity of semiconductors can great enhanced due to the bandgap changes on inter-atomic spacing. However, limited by the stretchability and wearability, metal and bulk semiconductor-based strain sensors are not suitable for flexible strain sensors [171,172,173]. Thus, the conductive nanomaterials with flexible polymers we discussed in Section 3 were employed for flexible piezoresistive sensors. For example, PDMS encapsulated carbonized phenol formaldehyde foam for flexible and multipurpose piezoresistive sensor was fabricated and capable of accurately monitoring a subtle bending strain of 0.05% [174]. Nevertheless, the lower GF value of 20.5 restricts its further applications. And this weakness is very prevalent among the piezoresistive sensors caused by the inferior interfacial adhesion strength and mismatch of elasticity between conductive nanomaterials and stretchable polymer matrix. Concerned with this issue, utilizing the microstructure engineering strategy was proposed to enhance the sensitivity and overall property of the flexible sensors.

### 4.3. Disconnection Mechanism and Microcrack Propagation

The electrically conductive paths distributed in the polymer matrix commonly forming overlapped networks for better conductivity. Forced by the applied external strain, the deformation of the material led to the decreased overlapped area and interconnected access, which finally resulted in the increased resistance. For example, a disconnection mechanism could be easily observed among metal NWs networks and graphene flakes-based strain sensors [175]. With a more visual explanation, the schematic illustration with a modeling of percolation networks under strains were investigated, materials including AgNWs [91,122], AgNP [116], and graphene flakes [176] embedded with polymers. Demonstrated by those graphic schematic and microstructural characterization, simultaneously assisted by computational mode analyzation, the complete structural changes have been revealed in a straightforward and distinct way. The irreversible electrical signal caused by inside slides between conductive nanomaterials and stretchable matrices during this process is also an issue worthy to be concerned with. Another disconnection structure responded to strain, such as the spring-like structures, were also discovered [177].

Different with disconnection mechanism, when certain strains applied to sensors, the intrinsic or derived fatigue microcracks generated and propagated internally which brought the change of electrical signals. When the strain is removed, the microcracks restore to its initial position, although some irreversible features developed. By using microcrack propagation mechanism, ultrasensitive graphene-on-polymer strain sensors were prepared with ultrahigh GF of ~10^3^ under 2~6% strains and ~10^6^ under higher strains [46]. The woven mesh configuration and high-density cracks, which finally leading to the fracture of the materials, are attributed to its high performance. However, the limitary strain of 8% weakened its further application. For that matter, we have proposed a crack-based nickel@graphene-wrapped polyurethane sponge ternary hybrid for highly stretchable and ultra-sensitive strain sensor (stretch to 65%, GF of 36.03~3360.09) [95]. The microcracks generated from the electrodeposition process and propagated by the applied strain. Interestingly, the double microcracks mechanism was also essential for high GF value. For disconnection and microcrack propagation mechanisms, the balance between sensitivity and linearity for sensors is an important point of further consideration for the researchers.

### 4.4. Tunneling Effect

When a nonconductive barrier exists between the closely spaced adjacent nanomaterials, the electrons can tunnel through with a complete pathway and this phenomenon called tunneling effect. According to the Simmons’s theory, [178] the electric resistance under tunneling effect can be calculated by the equation of $R_{\mathrm{tunnel}}=\left(\frac{L}{N}\right)(\frac{8\pi \mathrm{hs}}{3\gamma Ae^{2}})\exp\left[\gamma s\right]$, while $\gamma =\frac{4\pi \sqrt{2m\varphi }}{h}$, where L is the number of particles forming a single conductive path, N the number of conducting paths, h the Plank’s constant, s the least distance between conductive particles, A the effective cross-section area, e the electron charge, m the electron mass, and φ the height of potential barrier between adjacent particles. Tunneling effect was observed between the conductive nanomaterials and polymers, such as graphene, [179,180] CNTs, [181,182] AgNWs [91], and PDMS [183]. The nonconductive barrier distance, also known as cut-off tunneling distance, is related to the type of conductive nanomaterials and insulating media, as well as the fabrication approaches. It is reported that the nonconductive barrier critical distance for AgNW–PDMS–AgNW, CNT–polymer–CNT, and paralleled graphene sheets-polymers-graphene sheets are 0.58, 1–1.8, and 2–3nm, respectively [91,184,185,186,187].

For flexible strain sensors fabrication, tunneling effect plays a vital role to verify the experimental results and predict the mechanism model, according to the inside microstructures. Fu et al. reported CB and multiwalled CNTs (MWNTs) mixture combined with thermoplastic PU for strain sensors with tunable sensitivity [182]. By the modeling study of tunneling theory for the working mechanism explanation, they found the high consistency result of the theoretical model and the experimental data. Analogously, CB/MWNTs/polyvinylidene fluoride-hexafluoropropylene composite films were fabricated as strain sensors [181]. Through the analysis of conductive nanofillers by tunneling effect, the improved sensitivity and alleviated nonlinearity of the sensors were attributed by the content of CB and MWNTs, which have manifested instructive significance of the modeling method.

## 5. Applications

**Figure 6:**
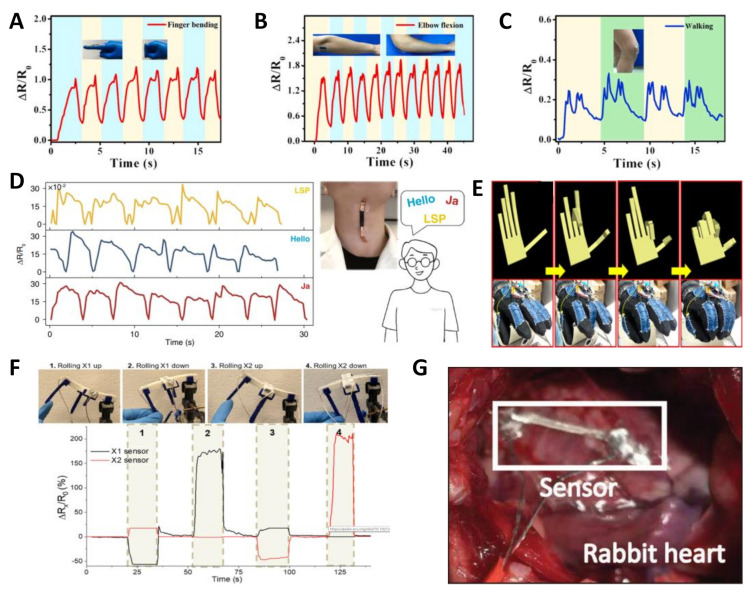
(**A**–**C**) The application for strain sensor used in human motion detection (finger bending and releasing, elbow flexion, walking). Reproduced with the permission from [74], copyright the Elsevier B.V., 2020. (**D**) Sensing application of the strain sensor when monitoring speaking different words with sample fixing at neck. Reproduced with the permission from [188], copyright the Springer Nature, 2021. (**E**) Control of avatar fingers in the virtual environment using wireless smart glove system. Reproduced with the permission from [91], copyright the ACS Publications, 2014. (**F**) Four types of actuations were applied to the actuator, proving the capability of multidirectional bending detection. Reproduced with the permission from [11], copyright the ACS Publications, 2020. (**G**) The fabricated sensor on the surface of a beating heart of a rabbit by the liquid-metal-based cardiac patch. Reproduced with the permission from [189], copyright the Wiley Online Library, 2019.

Based on the ingenious design and distinguished performance of the reported flexible strain sensors, there are a great deal of applications that have been demonstrated for various occasions. In this section, the in vitro detections for human health and implantable in vivo devices for biomedical engineering, as well as human-machine interface and soft robotics applications are reviewed (Figure 6).

### 5.1. Human Motion Detection and Biomedical Health Monitoring

The variation of electrical signals against the applied strain of the flexible strain sensors brings plenty of applications on wearable and skin-mountable electronics from the body, top to bottom. Figure 6 shows the reported sensors demonstrated in different parts of the human body. For monitoring eyeball movement, porous graphene fibers have been demonstrated and moved into a gauze as strain sensors for the detection of eyeball rotation and eyelid blinking [190]. Our group proposed a highly sensitive strain sensor for sensing of facial muscle stretch [95]. In addition, other low strain motions, such as swallowing, [134,179,191] voice vibrations, [164,192,193] pulsing, [29,135] blowing, [194,195] and neck joint motion [196,197] biomedical test have also been presented by different composites. On the other hand, large strain movements, including finger [198,199] and knee bending [200,201], wrist [60] and elbow motions [202,203], plantar distribution [204], respiration rate [205], and some derived sports behaviors requires high stretchability and better sensitivity of the sensors. For example, a strain sensor with multidirectional sensing capability and high GF value of 180 has been introduced based on carbon nanofiber-PDMS composites [206]. The highly aligned structure of carbon nanofibers exhibited different morphological changes and distinct electrical resistance changes in different loading directions. Besides those applications on human body, the growth of a bamboo plant was monitored using a PEDOT:PSS-based strain sensor. The grew up by 2 cm during the 4 days monitoring of bamboo was studied, which subtly revealed the higher growth rate at night and peaked at midnight. Generally, combined with signal processing and output circuit on flexible board, the real-time monitoring of human motions for evaluating of sport performances and biomedical demonstrations of wearable and skin-mountable electronics show tremendous application potential in the future.

### 5.2. Implantable Devices

For implantable electronics and devices, higher standards are required due to the complicated and variable internal environment. Stability, non-poisonous, biocompatible, flexibility, and conformity are some basic characters for bioinert implant strain sensors. Toward real-time monitoring of bladder volume, a novel soft conductor (gold-coated titanium dioxide nanowire layers embedded in an ultrasoft silicone elastomer) with high cyclic stability and a strain sensor with a chipless wireless readout was presented by János Vörös’ group [207]. The mechanical properties of designed strain sensing elements are similar with the tissue, which is vital to avoid interference signals. Additionally, the distinct linearity was observed when the bladder volume above 95 mL. Besides, a buckled sheath–core fiber composed of CNTs and thermal plastic elastomer was reported as an implantable device, which attached on the tendon of a lab rat for real-time quantitative assessment of tendon rehabilitation [208]. By calculating the defined angle, the sensing signals versus variation of the angle reflect the loaded force from the muscle to the ankle, which is helpful to guide rehabilitation training.

In addition to the above-mentioned implantable applications, cardiac health detection and time treatment are other key missions for flexible electronics and devices. To this end, great efforts have been made seeking effective approaches and materials. In order to calculate the pressure between 3D multifunctional integumentary membranes (3D-MIM) and the heart with arbitrary shape for organs, a universal and easy-to-use model was established by Su et al. [209] They found the average pressure is insensitive to the frication and delamination between 3D-MIM and the heart by finite element analysis (Figure 6). Park et al. developed an epicardial mesh consisted of ligand exchanged AgNWs and Styrene-butadiene-styrene (SBS) rubber to resemble the innate cardiac tissue and confer cardiac conduction system function, to enable electromechanical cardioplasty [210]. The excellent contractility and conductivity ensured the reliable electrical signal detection and rational electrical shock. Through theoretical estimation and practical test on rat heart, the authors proved the improved systolic function without impeded ventricular relaxations. Besides, Au coated epicardial mesh showed the same effective functions and biocompatibility. Also for this purpose, other 3D-MIMs were proposed by using different approaches (such as 3D printing [211]) and materials (such as Ag-Au core-sheath nanowire composite [117]), which were multi-functional electronics. Overall, the further development of flexible sensing materials will promote greater development of the implantable electronics and devices with real-time monitoring and immediate treatment beyond functions.

### 5.3. Human-Machine Interface and Virtual Reality Technology

Flexible strain sensors could be able to detect the finger bending based on the changes of electrical signals. By further development of human-machine interfaces, the changed electrical signals can be transferred to smart devices or robots, and synchronously control the actions of that equipment. For example, a smart glove system was fabricated by integrating AuNWs/PANI strain sensors with wireless devices [110]. The remote control of a robotic arm to pick up and put down the target object kept the risks away from people. Recently, a group of tactile sensors using piezoresistive foams was proposed and mounted to each finger of the 3D printed gripper, [212] the state of each sensor was displayed by virtual LED when the gripper works and releasing. Moreover, combined with the concept of “second skin” for human motor assistance raised by Eugene C. Goldfield et al., [213] those reported sensors could be widely carried forward in the field of soft robots and remote manipulation requirement. For emerged virtual reality technology, flexible strain sensors were utilized as an interactive drive to control the finger motion of an avatar in the computer virtual environment [91]. As shown in Figure 6, the synchronized actions of finger bending were achieved in reality and virtual environment. This technology is beneficial to rehabilitation engineering and entertainment demand.

## 6. Manufacturing Approaches

The long-range prospects for the flexible strain sensors require massive production industry in combination with specific applications. However, traditional manual fabrication operation in laboratories limited by low efficiency and poor repeatability. Thus, developing a controllable and reliable manufacture method is desired by the rapid expansion of flexible electronics. In this section, we introduce three categories of recently developed, promising fabrication methods for flexible strain sensors.

### 6.1. Printing Technology and Biomimetic Methods

**Figure 7:**
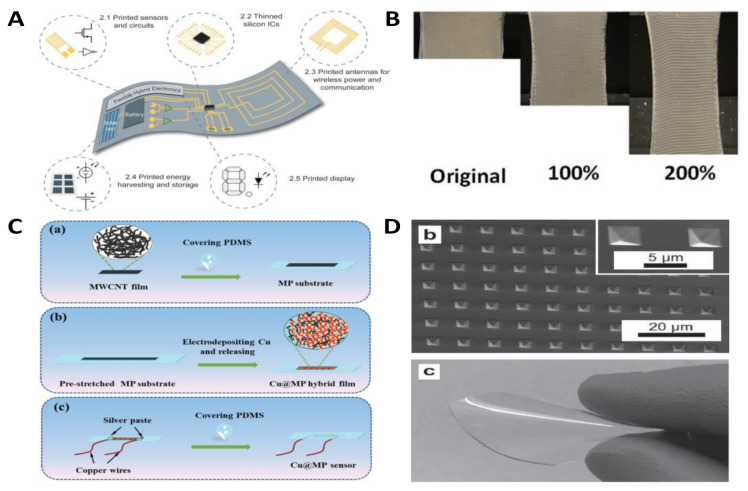
(**A**) A flexible electronic system, showing the key components of flexible hybrid electronics. Reproduced with the permission from [214], copyright the Wiley Online Library, 2020. (**B**) Photographs of the sensor network fabricated by electrospinning process under 0%, 100%, and 200% strain. Reproduced with the permission from [215], copyright the Wiley Online Library, 2020. (**C**) Schematic illustration of the fabrication process of strain sensor by electrodeposition process. Reproduced with the permission from [216], copyright the Royal Society of Chemistry, 2020. (**D**) SEM images of the microstructure prepared by micro-nano machining technology on flexible substrate and the image of the elastomer film. Reproduced with the permission from [217], copyright the Wiley Online Library, 2015.

Printing technology, originated in China, is one of the four great inventions and a milestone in human progress. Now, the various applications of this traditional and practical printing technology in today’s society, have caused important changes in different aspects especially when required large-scale production. For the rapid and massive production of flexible electronics, recently, printable flexible electronics have drawn much attention, due to the advantages of printing technology including low manufacture cost, controllable processing steps, and personalized customized production way (Figure 7).

According to the classification of printing technology, the non-contact printing techniques, such as screen printing and inkjet printing processes, have been widely used for flexible strain sensors because of their respective merits [218,219]. With the advantages of simplicity and adaptability, screen printing as one matured technology has been practiced in flexible electronics extensively based on various substrates. To explore the textile based capacitive sensors by screen printing technology, Josue Ferri et al. provided a guideline to choose the materials and printing parameters, and reached a limited result with capacities of less than 60 pF [220]. Besides, Han-Ki Kim’s group reported a screen-printed AgNPs on PU substrate for electrodes and sensors, which had better conductivity and stretchability. Although great progresses have been achieved on screen printing technology, the low accuracy when it requires tiny line width needs to be optimized [221]. To achieve high-quality and high-resolution printing patterns, inkjet printing technology emerged rapidly in recent years due to the high degree of accuracy, ecofriendly, and low-cost manufacture process, as well as facile controlled parameters. On the one hand, multi- types and functions printable inks with reliable properties (e.g., biocompatibility and low-temperature processability for wearable electronics) were developed for a higher standard inkjet printing process. A highly stable and sensitive sensor based on hybrid conductive network that consists of layered double hydroxides and AgNWs was reported, [222] which could be prepared by inkjet printing technology. In other instances, the advancing strategies run to minimized size and uniform high-resolution details were introduced by various approaches on inkjet printing method [172,223]. Recently presented wearable inkjet-printed strain sensors for respiratory rate monitoring at different body postures has been derived and shows nearly identical distributions of reference respiratory rate sensors, [224,225] which makes it a promising and user-friendly option for clinical and daily uses.

With the development of advanced technology, the emerged 3D and four-dimension (4D) printed sensors have been rapidly developed due to their efficient manufacturing facilities, convenient and time-saving process, as well as programmable and intelligent properties (for 4D printing) [226,227]. Based on the development of those two multi-dimensional printing technologies, they are expected to be very promising methods for large-scale promotion. For 3D printing sensors, the printed sensing element, stretchable electrodes, and designed microstructure were reported based on different sensing mechanisms (piezoresistive, [228] piezoelectric, and capacitive). Another strategy is to integrate LMs with 3D printed channels for electrical connectivity [229]. Briefly summarized, remarkable scientific studies have been conducted in a 3D printing regard, however, further studies are required in search of reliable and cheap printable raw materials, as well as seeking methods for thin and smooth films and electronics. Based on 3D printing, 4D printing technology can realize the controllable changes in the shape, performance, and functions in terms of time and space dimension, so as to meet the application requirements of deformation, degeneration, and variable functions [230]. According to the reported works, coupled 4D printed structures (such as transformable structures [227]) with 3D printed, electrically functional devices (such as strain sensors) could be applied to control their shapes precisely. In addition, the 3D circuits can be efficiently fabricated combined with 4D printing technique. For example, Deng et al. reported 3D printing methacrylated macromonomers to fabricate shape memory objects that can be used in flexible and responsive electrical circuits [231]. Similarly, to further overcome the limitations (such as process, materials, and design) in the future, 4D printing technology can be widely used in the manufacturing of intelligent flexible sensors and wearable electronics.

Another vital fabrication technology is a biomimetic method, which includes learning active matters and architectures from nature to overcome the current problems and to develop new-type sensors. Inspired by the peculiar structures and functions, great advances have been made in the field of flexible strain sensors [31]. To improve the sensitivity of sensors, one approach is to copy the mirco- and nanostructure of existing natural plants as the patterned sensing units on flexible substrates. For example, a flexible capacitive tactile sensor was prepared by utilize the bionic microstructures on natural lotus leaves by Zhang et al., [232] and it could be stretched to 33% strain with good sensitivity. In addition to the bionic plant, the octopus tentacles-, [233] nacre- [234] and duck web- [235] inspired biomimetic structures were reported for high performance flexible strain sensors. Niu et al. studied biological sensing mechanisms of the typical scorpion (Heterometrus petersii) in order to improve the sensitivity, [236] and the obtained crack arrays-based sensor exhibited ultra-high GF of 5888.89 upon 2% strain. In term of stretchability, an ultra-stretchable sensor with 1135% strain enhancement and 20,000 cycles stability based on buckled sheath–core fiber was reported [208]. Moreover, by mimicking human skin structure, a fast response (<10 ms) E-skin sensor with low detection limit (0.6 Pa) was fabricated combining two layers of patterned CNT-PDMS composite films [237]. Besides the above-mentioned features, more smart functions imitated from human skin and extended, were endowed with flexible strain sensors, such as self-healing, superhydrophobicity, and biodegradability, etc. The skin-like multi-functionalized sensor is the face of the future, therefore, Wang et al. presented a skin-inspired, highly conformable, and flexible sensor array with in-plane strain, pressure, temperature, humidity, light, and magnetic field sensing functions, [238] which is an important leap in the flexible and wearable electronics filed.

### 6.2. Micro-Nano Machining Technology

Traditionally, Micro- and Nano- fabrication technology is widely applied to the rigid substrate-(such as silicon and gallium nitride substrate) based fabrication process of electronic components integration. When flexible electronics meet this machining method, the advantages can be presented to a great extent after overcoming the current difficulties. It is believed that micro- and nano- fabrication technology occupy a decisive position to promote efficient and scalable manufacturing of multi-functional flexible electronics and integrated devices in the near future. Therefore, in this part, we review recent progress of flexible strain sensors and devices that are machined by nanoimprint lithography (NIL), laser technique and hybrid system methods.

As the most successful and predominant nano-patterning technique, which equipped the most feature sizes scaled down to a few nanometers across, photolithography on rigid substrates technology is not suitable for flexible substrate-based electronics. Different from photolithography, NIL provides an efficient strategy for high resolution nano-patterning production. By using NIL, micro/nanostructures were prepared for flexible sensors, and those sensors usually exhibit higher sensitivity, faster response time and lower hysteresis compared with bulk film-based sensors. For example, a resistive-type strain sensor (300 μm in width) with good linearity and robust stability was introduced based on micro molding and transfer printing process by Kim et al. [239] In order to reach to higher sensitivity, Bao et al. introduced the highly uniformed microstructures made with an imprinting process, the obtained sensor shows a low detection limit of 5 mg with degradable and biocompatible properties [217]. Besides, Nie et al. reported a strain sensor that shows high sensitivity with a GF of 1140 and elevated optical transparency of 87% by micro molding and then embedded multiwall CNTs into PDMS microtrenches [240]. Those micro- and nanostructures-based flexible sensors by NIL technology have speeded up the development and major expansion of wearable and flexible electronics for human health monitoring and soft robot applications.

Laser processes provide another approach for patterned, ultrathin and large-area manufacturing flexible electronics. Recently, the laser lift-off, laser-assisted printing, and laser-assisted transfer printing techniques are extensively researched in the flexible electronics field. Laser lift-off can be used as a non-contacting substrate transfer process for releasing the flexible film from the transparent substrate for the upper prefabricated device. For example, highly sensitive flexible motion sensors consisting of gallium nitride piezoelectric nanogenerators and light emitting diodes were fabricated utilizing the laser lift-off process, [241] which exhibits good recognizability for bending and strain motions. To investigate the mechanism and technologic characteristics of laser lift-off process of polyimide film, Huang et al. presented a flexible strain sensor on ultrathin PI film (2 μm) after being released from the glass substrate without any damage and wrinkle [242]. Additionally, this study provides an attractive route to optimize the laser lift-off process for large-scale production of ultra-thin flexible electronics. Laser-assisted printing technique enable the depositing and patterning of various functional materials on flexible substrate directly with material compatibility, and controllable process and feature size. For instance, Suganuma et al. reported a 100% AgNWs-based stretchable and transparent electrode with a width of 200 μm and excellent conductivity by laser-induced forward transfer technique [243]. Laser-assisted transfer printing technique is a good candidate of non-contract transfer printing process with high selectivity, versatility, and scalability for flexible electronics. Additionally, the typical examples for that are laser-assisted die transfer process, laser-driven micro-transfer placement process, and laser-driven shape memory effect for transfer printing. In addition to the above-mentioned three types of laser-based electronics, there are some laser-induced been carbonized and reduced materials have been applied to flexible strain sensors. For example, to prepare degradable multimodal sensor, transferring laser-induced porous carbon from PI to the starch film was conducted by Huang’s group [244]. The resulted strain sensor shows multi-sensing functions, including strain, temperature and pressure. In order to get a highly responsive flexible strain sensor (high GF value of 725), Yu et al. demonstrated laser-scribe thermal reduction induced polystyrene nanoparticle doped reduced graphene oxide composites for human health monitoring [245].

Actually, there are a lot of flexible strain sensors fabricated by mixed the above illustrated preparation methods, here we name those “hybrid system methods”. By centralizing the strength of individual methods, the obtained sensors possess excellent performance. For example, Ren’s group developed an artificial graphene throat by laser-scribed graphene and water-assisted transferring process [246]. Through depth research on graphene-based sound detection and sound emission, this sensitive sensor is very meaningful for helping mute people to “spear” in the future. For continuous and non-invasive intraocular pressure monitoring, by employ photolithography, CVD, and thermoforming methods, Ren et al. fabricated a wireless monitoring system with 85% transparency for diagnosis and treatment of glaucoma [247]. Scalable fabrication of flexible electronics is essential in the future, for this purpose, Lin et al. proposed an innovative design of 3D structures for flexible electronics sensor combined photolithography with screen printing technology, [248] which contains 25 × 25 array of sensing pads and bumps within 15 × 15 mm^2^ area. Thus, as chapter summary, micro-nano machining technology is an efficient and controllable tool for large-scale and low-cost production of flexible strain sensors and electronics. Combining with other steerable techniques and materials, we believe that it will plays the decisive role in the sustainable rapid development of integrated flexible devices.

### 6.3. Electrospinning and Electrochemical Technology

Electrospinning as an important fabrication technology, which also aroused much attention due to obtained fibers with some outstanding properties, such as high specific surface area, tunable surface morphologies, good mechanical flexibility/strength, and facile surface functional decorating. By weaving the electrospun fibers, smart textiles and clothing can bring more functions for the wearer. In addition, electrochemical treatment is another approach for the fabrication of flexible strain sensors. Therefore, the development of electrospinning and electrochemical technology applied to flexible strain sensor and electronics are focused in this section.

As one of the most convenient ways to produce nanofibers, electrospinning technology possesses unique advantage in flexible strain sensors and electronics fields by low-cost and scalable manufacturing mode. The strong piezoelectricity of electrospun polyvinylidene fluoride (PVDF) nanofiber membranes make it a suitable choice for sensors. For example, Persano et al. introduced a large area, flexible piezoelectric material that consists of sheets of electrospun fibres of the polymer poly(vinylidenefluoride-co-trifluoroethylene) [249]. The prepared material offers excellent piezoelectric characteristics and ultra-high sensitivity even at small pressure (0.1 Pa). Based on this work, to design a tactile sensor with high sensitivity and shape adaptability, Ding et al. developed a piezoelectric response of sensing layer by coaxial electrospinning, [250] which largely was enhanced through the synergistic effect of barium titanate nanoparticles and graphene oxide nanosheets in a hybrid coaxial structure. Besides the highly shape adaptive without sacrificing its sensing capability, the mapping sensors were capable of sensitively discriminating the flexion and extension of various synovial joints. By microstructure design, hollow structure PVDF fibers were obtained by optimal electrical field and repolarization process [251]. In addition to PVDF, other electrospun polymers were also investigated for high performance strain sensor. For instance, anisotropic resistive-type flexible strain sensors based on PU have been developed by Zhao’s group by near-field electrospinning method [215]. Through the theoretical modeling and tuned geometry of the PU grid and concentration of the sprayed AgNWs, high GF value of 338.47 and high stretchability to 200% strain were resulted by the sensors. The strengths of electrospinning technology in flexible electronics have been fully demonstrated in recent years, however, the variety of mixed conductive electrospinning solutions, the fabrication of superfine fiber, and the degree of technical integration with other technologies are still needed for further advancement in the future.

Electrochemical method can provide conductive decorating (such as metals and conductive polymers) during the fabrication process of flexible strain sensors. To further improve the sensitivity of stretchable strain sensors, we prepared a resistive-type sensor based on electrodeposited nickel on graphene wrapped PU sponge and “double micro-crack mechanism” [95]. The highly sensitivity (GF value of 3360.09) and fast signal response ability (< 100 ms) make it suitable for wearable electronics. In addition, we also fabricated a highly stretchable electrode by electroless plating technique [252]. The 3D gold-nickel@graphene coated PU sponge exhibits excellent conductive stability when under 1000 cycles of stretching (up to 30%), bending, and twisting separately. Electrochemical decorated conductive polymers also researched for flexible strain sensor. For example, a strain sensor composed of PDMS and PANI was fabricated by electrodeposition method for PANI preparation [56]. The electrodeposited PANI and micro-cracks based sensor shows high sensitivity. Carli et al. developed neural recording and stimulation electrodes by electrodeposited PEDOT:Nafion, [253] and this material has great application potential in biomedical engineering. It is observed that the concomitance generated micro-cracks during electrodeposited process and strain-induced extended cracks contributing a great deal to the high sensitivity of the strain sensors. However, to achieve controllable micro-crack fabrication for adjusted sensitivity to adapt to different application situations still remains a challenge.

## 7. Conclusions and Outlook

Flexible and wearable strain sensors have grown up quickly due to the breakthrough in the limitation of conventional metallic foil or semiconductor-based strain sensors. The excellent flexibility and stretchability contributes outstanding conformity and anti-jamming capability to skin-mountable and wearable applications. In the meantime, the fast advancement of material science and micro-/nano- electronics technology boost the further applications of flexible strain sensors for real-time health and motion monitoring, home rehabilitation, man-machine interface, and soft robot. In this review, we have presented a comprehensive summary on the latest development of the flexible and wearable strain sensors, including the critical evaluation index, materials developments, sensing mechanism, computational simulation analysis, applications, and manufacturing approaches. Besides the frequently used nanomaterials, during the last decade, more advanced materials were introduced for sensors preparation, such as liquid metal, third generation semiconductor materials, 2D MXene materials, etc. Based on various sensing mechanisms and further improved performance, the functions were extended with more integrated devices, while the applications expanded from skin-/robot skin- mounted to implantable monitoring and stimulation. In addition, more advanced manufacturing technologies with efficient and scalable capability were employed for controllable and large-area fabrication of flexible strain sensors, which further facilitates the generalized application of that.

However, there are still many challenges need to be addressed. Firstly, there are some points that should be indicated on the materials aspect. The Young’s modulus difference between nano-conductive materials and elastomer polymers can cause the weak adhesion, which is one important factor resulting the failure and instability for the strain sensors. The poor stretchability and conductivity of conductive polymer limits its further application. Secondly, based on the developed materials and the obtained performance, controllable fabrication of desired sensors for different application scenarios is necessary, such as high sensitivity under micro-/small strain, high stretchability for animated movement, and stable linearity during the usable range applicability. Thirdly, to cooperate the integrated monitoring system, the portable and even implantable flexible power management circuits and wireless read out module need to be developed for long-term and accurate monitoring. Fourthly, the innovations in device structure should be realized to generate sufficient power to extend the operating cycle of various wearable devices. For flexible strain sensor devices, the miniaturization of integrated sensor arrays with multiple sensing modes is essential to eliminate signal crosstalk and distinguish various external stimuli. Finally, combing the big data platform, further research should focus on large scale and low-cost manufacturing technology for strain sensors-based multifunctional system for extended applications. Thus, interdisciplinary crossover research activities will be the notable trend for flexible and wearable strain sensors in the future.

## Figures and Tables

**Table 1:** Summary of performance results of recently reported stretchable strain sensors.

Materials	Sensing Mechanism	Stretchability (%)	Gauge Factor	Linearity	Response Time (ms)
Graphene-PU [150]	Resistive	100	11–80	Linear up to 15%	200
Graphene foam-AgNWs-PU [151]	Resistive	60	11.8	Nonlinear	40
Graphene Belts-dragon skin [152]	Resistive	55.55	175.16–13278	Linear up to 35%	120
Graphene-PDMS [153]	Capacitive	80	0.98	Linear	180
Carbon nanofibers/AgNWs-PDMS [154]	Capacitive and piezoresistive	50	2.29–8.21 for two facesheets 0.81 for sensor	Linear	130–150
MWCNTs-PDMS [155]	Resistive	40	3.89–7.22	Linear up to 20%	-
CNTs-PDMS [156]	Resistive	44	0.4–22.6	Linear up to 20%	-
Metallic CNTs-PDMS [157]	Resistive	30	-	linear	-
CNTs-elastic bands- polydopamine [158]	Resistive	920	129	Nonlinear	220
CBs–PDMS [159]	Resistive	30	29.1	Linear	-
CBs-nitrile butadiene rubber-polydopamine [28]	Resistive	180	346	Nonlinear	-
AuNWs-AgNWs-PDMS [113]	Resistive	90	12–2370	Nonlinear	-
AgNWs-AuNWs- PDMS [160]	Resistive	70	236	Nonlinear	-
Self-healing polymer-AgNWs/-PDMS [161]	Resistive	60	1.5	Nonlinear	-
AgNWs–Ecoflex [162]	Capacitive	50	0.7	Linear	40
AgNWs-CBs-TPU [163]	Resistive	100	21.12	Three linear regions	-
AgNPs-graphene-TPU [164]	Resistive	1000	7–476	Nonlinear	-
Platinum (Pt)–PDMS [165]	Resistive	2	2000	Nonlinear	-
PVA-PEDOT:PSS-PDMS [166]	Resistive	30	14–110	Four linear regions	40
Liquid metal-PDMS [167]	Resistive	13.3	3.53	Linear	-
Ti_3_C_2_Tx MXene/CNTs [130]	Resistive	130	4.4–772.6	Nonlinear	-
SiC-Ecoflex [135]	Resistive	<5%	Up to 247020.2	Nonlinear	200

## Data Availability

Data sharing not applicable.
